# Exploration of calcium and amino acids for children with primary cardiomyopathies based on genetic characteristics

**DOI:** 10.3389/fped.2025.1631632

**Published:** 2025-11-18

**Authors:** Shirui Jiang, Ailin Zhang, Jiegang Deng, Wei Wang, Jingyu Wang, Hongyu Chen, Liqin Zhu, Wei Liu

**Affiliations:** 1 First Central Hospital of Tianjin Medical University, Tianjin, China; 2 Tianjin Children’s Hospital, Children’s Hospital, Tianjin University, Tianjin, China; 3 Precision Medicine Laboratory, Precision Medicine Center, Tianjin Children’s Hospital, Children’s Hospital, Tianjin University, Tianjin, China; 4 Tianjin University of Traditional Chinese Medicine, Tianjin, China; 5 Department of Pharmacy, Tianjin First Central Hospital, Tianjin, China

**Keywords:** pediatric primary cardiomyopathies, mutated genes, calcium, amino acids, exploration

## Abstract

**Background:**

Pediatric primary cardiomyopathies (PCMs) are rare diseases with complex causes and nonspecific treatment. The influence of electrolytes and amino acids (AAs) on cardiomyopathies has not been extensively studied. This study aimed to explore clinical characteristics and the usage of electrolytes and AAs in children with PCMs.

**Methods:**

Children diagnosed with PCMs who had genetic test reports were included. Relevant information was collected and processed, and clinical characteristics and mutated genes were clarified. Gene databases were searched to explore related electrolytes and AAs in the treatment of PCMs. The effect of calcium was explored in children with DCM. Paired samples T tests and nonparametric Wilcoxon signed-rank tests were performed for comparison between before and after using calcium.

**Results:**

In this study, 27 children with gene test results were enrolled to perform gene-related analysis. The median age was 2.5 years old. Mutated genes were collected, including pathogenic, likely pathogenic, uncertain significance, and other mutations. The most frequently mutated genes related to dilated cardiomyopathy (DCM) were *TTN*, *MYH7*, *NEXN*, *TNNI3*, and *SCN5A*. In hypertrophic cardiomyopathy (HCM), *MYBPC3*, *MYH7*, *PRKAG2*, *RAF1*, and *RBM20* were prevalent. Calcium and AAs (serine, cysteine, arginine, tyrosine, and alanine) were related to the mutated genes detected in children with PCMs. In addition, 17 children treated with calcium showed significant improvement in heart function.

**Conclusions:**

For children with DCM, calcium supplements may be beneficial. AAs, including serine, cysteine, and arginine, could be used for supplementary treatment in children with DCM and HCM.

## 1 Introduction

In the statement of the American Heart Association, cardiomyopathies (CMs) are rare in children, with high mortality (1). Causes are heterogeneous in pediatric CMs, ranging from genetic mutations to systemic diseases. The classifications of CMs are complicated and varied. According to the World Health Organization, CMs are classified into primary and secondary. Primary cardiomyopathies (PCMs) are heart muscle diseases in the absence of secondary pathogenic causes. Based on morphologic abnormalities and pathophysiology, PCMs were grouped into dilated cardiomyopathy (DCM), hypertrophic cardiomyopathy (HCM), restrictive cardiomyopathy (RCM), and arrhythmogenic right ventricular cardiomyopathy (ARVC) (2). According to the European Society of Cardiology in 2023, left ventricular noncompaction cardiomyopathy (LVNC) was also categorized as a type of CMs characterized by unique changes in morphology and function of the left ventricle (3).

DCM is defined as left ventricular (LV) dilatation and systolic dysfunction in the absence of abnormal loading conditions and coronary artery disease (4). In DCM, common direct causes are gene mutations, infections, and autoimmunity (5). Many genes are associated with pediatric DCM, such as *TTN*, *MYH7*, *RBM20*, *TNNT2*, and *DSP* (6). HCM is a primary inherited cardiac disease characterized by LV hypertrophy without the existence of abnormal loading factors (e.g., congenital heart diseases, hypertension) (8, 9). *TTN*, *MYH7*, *MYH6*, *PRKAG2*, and *MYBPC3* are related to pediatric HCM (6). RCM, ARVC, and LVNC are rare in CMs. RCM is described as a myocardial disorder characterized by structural and functional abnormalities, with the characteristics of restrictive ventricular filling (9, 10). LVNC, also called LV noncompaction, is characterized by excessive trabeculation in the left ventricle (11, 12). ARVC is a right ventricular (RV) inherited CM. Mutations of the gene encoding sarcomeric or cytoskeletal proteins are the common causes of RCM and LVNC (9, 11). Mutations of genes encoding desmosome proteins play a key role in the pathogenesis of ARVC (13).

Due to the rarity and high mortality, the research related to PCMs in children was limited. In addition, clinical manifestations, disease progression, and drug reactions between children and adults are different. Therefore, it is essential to focus on the research of PCMs in children. At present, the treatment of pediatric CMs is empirical and symptomatic. For instance, medicines used for DCM are diuretics, cardiotonic drugs, vasodilators, and cardiovascular protective drugs (6). Despite the reliance on empirical approaches, little attention has been paid to investigating nutrients, such as electrolytes and amino acids (AAs). A few articles described the effect of electrolytes or AAs on pediatric CMs. For instance, children with hypocalcemic dilated cardiomyopathy could benefit from calcium supplementation (14). It was reported that serine and cysteine could exert cardioprotective effects (15, 16). This study aimed to explore the clinical characteristics of PCMs in children and the influence of appropriate supplementation of electrolytes and AAs to provide more references in the treatment of pediatric PCMs in clinical practice.

## 2 Methods

### 2.1 Study population

Children who had been diagnosed with PCMs and had gene test results in Tianjin Children's Hospital from 2019 to 2023 were enrolled. The detailed inclusion and exclusion criteria were provided in Supplementary Material S1.

The inclusion criteria were as follows: DCM was described as age and body surface area corrected left ventricular end-diastolic diameter (LVDD) higher than 112%, left ventricular ejection fraction (LVEF) lower than 45%, and/or left ventricular fractional shortening (LVFS) lower than 25% (1, 17). HCM was described as an increased LV wall thickness, represented by a higher mean ± two standard deviations (SD) (18). RCM was described as biatrial enlargement, normal ventricular diameter, and LV diastolic dysfunction (1). ARVC was mainly diagnosed by the revised task force criteria (19). LVNC was described as the ratio of non-compaction to compaction higher than two in the end-diastolic stage (20).

In addition, some secondary pathogenic characteristics were excluded. Cardiovascular factors, including hypertension, congenital heart disease, arrhythmia, and aortic stenosis, were excluded. Extracardiac factors such as thyroid or parathyroid diseases, infections, and maternal diabetes during pregnancy were also excluded (1, 17).

### 2.2 Data collection and statistical analysis

Demographic and PCMs characteristics such as age, gender, height, weight, type of PCMs, gene test results, laboratory examination, and imaging examination were collected. Normality tests were used for continuous variables. Normal continuous variables were presented by mean ± SD, and non-normal data were presented by the median and interquartile range (IQR).

### 2.3 Gene test, data processing and analysis

Peripheral blood genomic DNA was extracted using the QIAamp DNA Blood Mini Kit (Qiagen, Hidden, Germany). DNA quantification and purity testing were performed by Qubit DNA Assay Kit in the NanoDrop 2000 spectrophotometer (Thermo Scientific, USA). Genomic DNA was fragmented by Tagment DNA Enzyme 1 and then subjected to PCR amplification to construct a sequencing library. The Qubit 3.0 Fluorometer was used to detect library concentration. High-throughput sequencing was performed on the Illumina Novaseq 6000 sequencing platform, and the target for average sequencing depth was 200×. All data were compared to the reference sequence using the BWA algorithm. The ANNOVAR tool was used to annotate data. The Genome Analysis Toolkit was used to analyze gene variations. The 1,000genomes, ESP6500, HGMD, and other databases were used for screening and annotation. Tools such as SIFT and Polyphen-2 were used for bioinformatics analysis of missense mutations. Based on the frequency, function, and inheritance mode of various gene variations, literature, and other relevant information, the classification of mutated genes was conducted in accordance with the guidelines established by the American College of Medical Genetics.

Genecards and Drugbank are large public databases that integrate information from various databases and literature, which could list genes and their related electrolytes and AAs. PubMed and Web of Science were used to search for articles to explore the influence of calcium and AAs on CMs. The search keywords were “cardiomyopathy, dilated cardiomyopathy, hypertrophic cardiomyopathy, restrictive cardiomyopathy, arrhythmogenic right ventricular cardiomyopathy, left ventricular noncompaction cardiomyopathy, calcium, amino acids, serine, cysteine, arginine, alanine, and tyrosine”.

### 2.4 Preliminary exploration of calcium supplements

To evaluate the therapeutic effects of calcium, children with primary DCM admitted from 2019 to 2023 were included. All participants received calcium, and underwent echocardiography both before and after treatment. Three patients used oral calcium carbonate D3 (150–300 mg once a day), and 14 patients used oral calcium lactate (250 mg twice or three times a day, or 500 mg once or twice a day). Normality tests were performed to assess the distribution of data. Paired samples T tests and nonparametric Wilcoxon signed-rank tests were performed for comparison between before and after medication.

## 3 Results

### 3.1 Demographic and clinical characteristics

A total of 27 children were enrolled in this study. The demographic and clinical characteristics of children with PCMs were summarized in Tables 1, 2. The demographic and clinical details of each child were provided in Supplementary Table S1. Supplementary Table S2 was involved in the echocardiography indicators of DCM, HCM, and LVNC. Sixteen children were diagnosed with DCM, seven with HCM, and another four with RCM, ARVC, or LVNC.

**Table 1:** Demographic and clinical characteristics of patients with PCMs.

Variables	Patients with PCMs	Reference range
Age (year)	2.5 (0.6, 10.0)	0–18
Sex		None
Male, *n* (%)	17 (63.0%)	
Female, *n* (%)	10 (37.0%)	
Height (cm)	99.0 (72.0, 143.0)	None
Weight (kg)	13.70 (7.48, 35.90)	None
BMI (kg/m^2^)	15.94 (15.00, 17.84)	None
K (mmol/L)	4.53 ± 0.77	3.5–5.3
Na (mmol/L)	133.0 (128.0, 137.0)	137–147
Ca (mmol/L)	2.38 ± 0.18	2.25–2.75
Mg (mmol/L)	0.84 (0.76, 0.90)	0.70–0.91
Cl (mmol/L)	96.1 ± 6.2	99–110
CKMB (U/L)	13 (7, 21)	0–24
LDH (U/L)	276 (244, 348)	120–300
TnT (ng/mL)	0.030 (0.010, 0.061)	0–0.0223
Mb (ng/mL)		28–72
<28, *n* (%)	26 (61.9%)	
28–72, *n* (%)	9 (21.4%)	
≥72, *n* (%)	7 (16.7%)	
pro-BNP (pg/mL)	1,666.5 (351.1, 4,072.0)	0–132

PCMs, Primary cardiomyopathies; IQR, Interquartile range; SD, Standard deviation; BMI, Body mass index; CKMB, Creatine kinase isoenzyme; LDH, Lactic dehydrogenase; TnT, Troponin T; Mb, Myoglobin; pro-BNP, pro-Brain Natriuretic.

**Table 2:** The characteristics of echocardiogram indicators for patients with DCM, HCM, and LVNC.

Echocardiogram indicators	DCM	HCM	LVNC
IVS (mm)	4.8 ± 1.4	16.9 ± 4.1	4.0 (4.0, 4.0)
LVPW (mm)	5.7 ± 1.3	11.2 ± 4.5	3.8 ± 0.7
LVDD (mm)	41.2 ± 13.9	33.8 ± 5.5	36.4 ± 12.3
LVDS (mm)	32.0 (22.8, 41.0)	19.5 ± 2.7	27.0 (23.0, 35.0)
LVEF (%)	41.6 ± 13.9	72.8 ± 6.1	54.2 ± 9.0
LVFS (%)	20.0 (15.0, 24.0)	41.0 (40.0, 46.0)	27.0 ± 6.1

DCM, Dilated cardiomyopathy; HCM, Hypertrophic cardiomyopathy; LVNC, Left ventricular non-compaction; IVS, Interventricular septum; LVPW, Left ventricular posterior wall; LVDD, Left ventricular end-diastolic dimension; LVDS, Left ventricular end-systolic dimension; LVEF, Left ventricular ejection fraction; LVFS, Left ventricular fractional shortening.

The medians and IQRs of age were 2.5 (0.6, 10.0) years old. There were 17 males (63.0%) and 10 females (37.0%). Body mass index values were lower in these children, with a median and IQR of 15.94 (15.00, 17.84). Among the electrolytes, the median of Na^+^ (133.0 mmol/L) and the mean of Cl^−^ (96.1 mmol/L) were lower than the normal ranges. Compared with the normal range of 0–132 pg/mL, the pro-brain natriuretic peptide in these children was significantly elevated, with a value of 1,666.5 (351.1, 4,072.0) pg/mL. In children with DCM, the values of LVDD and left ventricular end-systolic dimension (LVDS) increased to 41.2 ± 13.9 mm and 32.0 (22.8, 41.0) mm, respectively. The LVEF was lower than the normal range, with a value of 41.6% ± 13.9%. In children with HCM, the interventricular septum and LV posterior wall exceeded the normal ranges, with values of 16.9 ± 4.1 mm and 11.2 ± 4.5 mm, respectively.

### 3.2 Mutated gene characteristics

In this study, mutated genes were collected and classified into four categories: pathogenic, likely pathogenic, uncertain significance, and other mutations. The mutated genes found in DCM, HCM, and LVNC are available in Supplementary Table S3. Among them, 28 types of genes were related to calcium and AAs.

**Figure 1:**
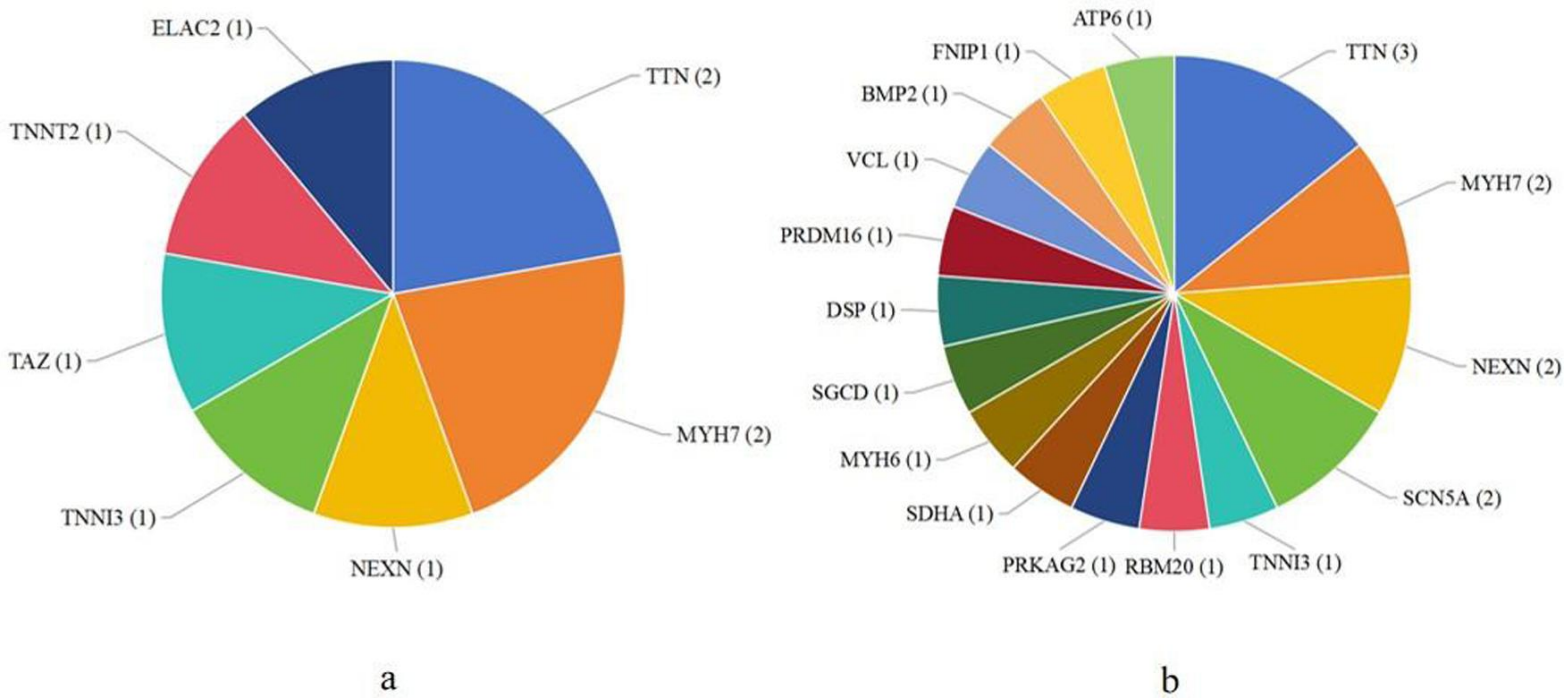
The number of patients related to mutated genes found in dilated cardiomyopathy. **(a)** The number of patients with pathogenic mutated genes; **(b)** The number of patients with likely pathogenic and uncertain significance mutated genes.

In children with DCM, seven genes were identified as pathogenic, such as *TTN*, *MYH7*, and *TNNT2* (Figure 1a). Sixteen genes were defined as likely pathogenic and of uncertain significance, such as *NEXN*, *SCN5A*, *TNNI3*, *MYH6*, *DSP*, and *VCL* (Figure 1b). There were also some other mutated genes, such as *TTN* and *JPH2*.

In children with HCM, three pathogenic mutated genes were detected, such as *MYH7*, *PRKAG2*, and *RAF1*. Fourteen likely pathogenic and uncertain significance mutated genes were identified, such as *MYBPC3*, *RBM20*, *MYH6*, and *SLC22A5*. *TTN* was also found as other mutated gene in HCM.

In children with RCM, *DES* was detected as a pathogenic mutated gene. In the likely pathogenic and/or uncertain significance mutated genes, two genes were found in children with ARVC, and 10 genes were found in children with LVNC.

### 3.3 Relationship between calcium and AAs and PCMs

Based on gene databases, calcium and 13 types of AAs were found to be associated with mutated genes in this study. Detailed information was provided in Table 3. A total of 21 genes were related to calcium. Among them, eight mutated genes (e.g., *TNNI3*, *TNNT2*) were found in DCM, whereas three mutated genes were found in HCM. *MYH7* and *MYH6* were found in both DCM and HCM. Additionally, *DES* was associated with RCM. Thirteen types of AAs were found, related to 22 genes. In genes related to DCM, the most frequent AAs were serine (e.g., *SCN5A*, *MYH7*), alanine (e.g., *SCN5A*, *TNNT2*), tyrosine (e.g., *DSP*, *VCL*), arginine (e.g., *SCN5A*, *MYH7*), and cysteine (e.g., *MYH7*, *VCL*). In genes related to HCM, arginine (e.g., *MYBPC3*, *MYH7*), tyrosine (e.g., *RAF1*, *SLC22A5*), serine (e.g., *MYH7*, *RAF1*), cysteine (e.g., *MYH7*, *RAF1*), and alanine (*RAF1*) were also found. Serine, cysteine, and tyrosine were found to be related to mutated genes in RCM, ARVC, and LVNC. *TTN*, a gene found in DCM, HCM, RCM, ARVC, and LVNC, was also related to serine and cysteine.

**Table 3:** Calcium, amino acids, and their related genes.

Calcium and AAs	Related genes
Calcium	*RYR1, RYR2, TNNI3, TNNT2, TPO, CDH2, HOMER2, TTN, MYH7, MYH6, SCN5A, DES, DSP, VCL, BMP2, KCNJ2, PLD1, GUSB, LAMB2, MYPN, JPH2*
Serine	*TTN, MYH7, RYR1, MYH6, SCN5A, DES, RAF1, ELAC2, DSP, VCL, BMP2, PLD1, APOB, CDH2*
Cystine	*TTN, MYH7, RYR1, DES, RAF1, VCL, BMP2, PLD1, APOB, CDH2, TPO*
Threonine	*TTN, MYH7, SCN5A, DES, RAF1, ELAC2, VCL, BMP2, PLD1*
Tyrosine	*GUSB, TPO, DES, RAF1, DSP, VCL, BMP2, KCNJ2, PLD1, SLC22A5, CDH2*
L-arginine	*SLC22A5, MYH7, RYR1, MYBPC3, MYH6, SCN5A, BMP2, KCNJ2, TPO*
Alanine	*RYR1, SCN5A, RAF1, TNNT2, BMP2, PLD1, APOB*
Glutamine	*MYH7, RYR1, SCN5A, RAF1, VCL, APOB*
Lysine	*TTN, SCN5A, BMP2, CDH2, TPO*
Proline	*TTN, RYR1, DES, VCL, ATP6*
Valine	*TTN, MYH7, SCN5A, RAF1*
Leucine	*MYH7, SCN5A, AKAP9*
L-glutamine	*RAF1, APOB, MYH7*
Asparagine	*MYH7, SCN5A, TPO*

AAs, amino acids.

The Genecards and Drugbank were screened for calcium and amino acids associated with mutated genes.

### 3.4 Influence of calcium on DCM

The details of children treated with calcium were listed in Supplementary Table S4. The comparison of echocardiographic parameters of DCM before and after using calcium was listed in Table 4. There were significant improvements in LVDD (*P* = 0.013), LVDS (*P* = 0.028), LVEF (*P* = 0.004), and LVFS (*P* = 0.013) after using calcium. The level of LVDD and LVDS decreased from 39.7 to 33.5 and from 33.5 to 29.0, respectively. The concentration level of LVEF and LVFS increased from 32.7 to 39.6 and from 15.3 to 18.5, respectively.

**Table 4:** Echocardiographic parameters for children with DCM treated with calcium.

Echocardiogram indicators	Calcium group (*n* = 17)		
	Before	After	*P*
LVDD	39.7 ± 10.1	33.5 (30.5, 38.0)	0.013
LVDS	33.5 ± 9.1	29.0 (22.5, 32.5)	0.028
LVEF	32.7 ± 9.9	39.6 ± 9.8	0.004
LVFS	15.3 ± 5.4	18.5 ± 5.1	0.013
LA	20.0 (18.0, 23.0)	19.0 (15.0, 22.0)	0.138

## 4 Discussion

The low incidence and poor progression of CMs result in challenging treatment. Although the medication used for pediatric CMs is similar to adults, such as beta-blockers and calcium channel blockers, the differences in causes, complications, and outcomes are significant between adults and children with CMs (6). In addition, due to the small sample size of pediatric PCMs and limited medication experience, reliable medication for pediatric PCMs is more difficult. Thus, it is significant to explore clinical manifestations and appropriate treatment methods for pediatric PCMs. This is the first study exploring the influence of electrolytes and AAs on pediatric PCMs.

In this study, most of the mutated genes were sarcomere protein genes. Some mutated genes were only found in one phenotype. For example, *TNNI3* and *TNNT2* were only found in DCM. *MYBPC3* and *RAF1* were only found in HCM. In addition, one mutated gene could also be associated with distinct phenotypes. For instance, *MYH7* and *PRKAG2* were found in DCM and HCM. *NEXN* was found in DCM and LVNC. *TTN* was found in DCM, HCM, RCM, ARVC, and LVNC.

The mechanisms of CMs induced by various gene mutations are as follows. *TTN* encodes titin, a giant sarcomere protein that functions as a significant component of the *Z*-disk (21, 22). In HCM, functional changes in myosin resulting from *TTN* mutation could increase the binding to α-actinin, whereas there is a decrease in DCM (21). Regarding *Z*-disk element mutations, several hypotheses indicated that HCM is characterized by a “stiff sarcomere”, whereas DCM is characterized by a “loose sarcomere”. Therefore, the binding among *Z*-disk elements in HCM and DCM will be tight and loose, respectively (21). *MYH7* encodes β-myosin heavy chain, which is an essential part of the sarcomere. In HCM, *MYH7* mutations are critical pathogenic causes. A hypothesis indicated that HCM is a sarcomere disease. It arises as a compensatory mechanism for reduced power generation caused by MYH7 mutations (21). In two cases with DCM linked to *MYH7* mutations, damaged sarcomere functions were observed (21, 23). Mutations of *TNNI3*, *TNNT2, RAF1*, and *NEXN* could be found in both DCM and HCM. *TNNI3* and *TNNT2* are common genes encoding sarcomere components, playing a critical role in Ca^2+^ regulation of muscle contraction and relaxation (24). *RAF1* encodes the component of the RAS-mitogen-activated protein kinases pathway, which plays crucial roles in myocardial biology (25). *NEXN* encodes nexilin, which is a protein of the *Z*-disk and contributes to its stability (26).

Most of our patients were detected with more than one mutation. It was reported that genetic causes of DCM could be divided into monogenic, polygenic, and multifactorial (e.g., environmental exposures) (5). There is an increasing recognition of polygenic mutations as the cause of HCM rather than monogenic.

Based on previous studies, calcium and some AAs were related to CMs (27–30). This study also detected that calcium and some types of AAs were associated with several genes of enrolled children. Therefore, supplements of calcium and these AAs may be potential therapeutic strategies for pediatric PCMs.

Calcium plays a key role in cardiac contraction coupling and electrophysiological signal conducting. Thus, defective intracellular calcium handling could result in contractile dysfunction, arrhythmia, and cellular hypertrophy (31). Mutated genes associated with sarcomere components (e.g., *MYH7*, *TNNT2*, and *TNNI3*) and *Z*-disk components (e.g., *TTN*) in HCM may result in increased Ca^2+^ sensitivity. Whereas, the situation may be contrary in DCM (21, 32). The mutations of *TNNI3* and *TNNT2* in DCM could reduce Ca^2+^ sensitivity and prolong reuptake time, resulting in lower Ca^2+^ concentration in the systolic stage and reduced myocardial contractility (32). Therefore, for children with DCM, calcium-based agents may serve as a potential therapeutic option. In our study, children given calcium expressed significant improvements in heart function. The average age of these children was one and a half years old. Older children treated with calcium showed relatively poor therapeutic effects from our limited information. Calcium supplements were suitable for younger children (less than or equal to one year old) with DCM. Of course, the effects of the base therapeutics regimen could not be ignored.

Additionally, the binding of Ca^2+^ to troponin C triggers actomyosin complex activation, contributing to sarcomere shortening and myocardium contraction (33). Increased Ca^2+^ sensitivity in HCM could increase sarcomere tension and excessive myocardial contraction (21). As a first-in-class selective cardiac myosin inhibitor for HCM, mavacamten reversibly combines with myosin ATPase and decreases activated myosin heads to increase actomyosin dissociation and make myocardium relaxation (34). Aficamten, a next-generation cardiac myosin inhibitor characterized by its shorter half-life and reduced drug-drug interaction, is currently undergoing clinical trials to investigate its potential role in HCM treatment (35).

This study also identified 13 AAs, which were associated with 22 genes detected in children with PCMs. Serine, cysteine, arginine, alanine, and tyrosine were related to genes found in both DCM and HCM. Serine and cysteine were also related to genes found in RCM, ARVC, and LVNC.

Some AAs have been reported to be beneficial to CMs. A study showed that the activated ATF4-dependent serine synthesis pathway could attenuate the DCM phenotype and improve systolic dysfunction by improving mitochondrial respiration and increasing levels of tricarboxylic acid cycle metabolites and ATP (16, 27, 36). In addition, the activated serine and one-carbon metabolism could reduce cardiac hypertrophy and improve ventricular function (37). It was reported that the activated serine and one-carbon pathways could reduce protein oxidation in mitochondria, retain ATP production, and prevent adverse ventricular remodeling in the context of myocardial hypertrophy (16). Additionally, some serine/threonine protein kinases may be beneficial for CMs. For instance, glycogen synthase kinase-3β could inhibit excessive myocardial contraction and suppress the expression of hypertrophy-related genes. Protein kinase G could reduce oxidative stress (38). Cysteine is involved in the synthesis of glutathione (GSH), which is integral to the endogenous protective system of the myocardium. It was reported that cysteine could stimulate the activity and expression of GSH peroxidase to protect against oxidative stress in myocardial cells (15). Furthermore, N-acetylcysteine, the metabolite of cysteine and precursor of GSH, could reverse hypertrophy and diastolic dysfunction in familial HCM (39). Arginine could produce NO, which plays a major role in protecting the myocardium by reducing inflammation in reperfusion and myocardial ischemia and maintaining myocardial contraction function (40, 41). In addition, Arginine could also eliminate oxygen radicals to protect the myocardium (41). Arginine has been suggested as a potential therapeutic option for mitochondrial CM and diabetic CM (42, 43). For this reason, arginine may be beneficial to pediatric PCMs, but the efficacy of arginine needs to be verified in clinical practice. Thus, children with PCMs may benefit from appropriate AA supplementation.

However, the link between cardiac function and two AAs, namely alanine and tyrosine, remains controversial. For example, alanine could exert a dual impact on heart function. On one hand, it was reported that levels of alanine were higher in patients with microvascular disease and lower in those with atherosclerosis (44, 45). On the other hand, it may be due to the inability to infer precise causality in observational studies. The increased level of alanine in CMs may be related to ischemic myocardium rather than being a risk factor for disorders (45). As for tyrosine, its oxidation products may lead to myocardial injury (46). However, another study also indicated that tyrosine could increase contractility in isolated atrial myocardium from patients with heart failure (47). Therefore, the influence of alanine and tyrosine in pediatric PCMs needs to be further studied.

This study has some limitations. Firstly, the current sample size is limited due to the rarity of pediatric PCMs. Larger samples are essential in the future to further explore the distribution of mutated genes in pediatric PCMs. Secondly, this is a single-center retrospective study, and bias in sample selection is unavoidable. Lastly, the influence of calcium and AAs in pediatric PCMs was inferred from genetic characteristics. The present study demonstrated that children who received calcium supplementation exhibited significant improvement in cardiac function. Of course, the efficacy of other potential drugs remains unexplored. Extensive preclinical and clinical validation studies are required to substantiate our findings.

## 5 Conclusion

Among the mutated genes detected in children with PCMs, 23 genes were found in DCM, while 19 genes were found in HCM. *TNNI3* and *TNNT2* were only found in DCM, whereas *MYBPC3* and *RAF1* were only found in HCM. Six types of mutated genes were observed in both DCM and HCM, such as *MYH7*, *RBM20*, and *PRKAG2*. Children with DCM, particularly those with a family history, may benefit from appropriate supplementation of calcium, serine, cysteine, and arginine to decrease morbidity, slow down the progression, and improve prognosis. Serine, cysteine, and arginine may also be beneficial to children with HCM. Additionally, serine and cysteine could also be applied as supplementary medication for children with RCM, LVNC, or ARVC.

## Data Availability

The original data included in this study are presented in Supplementary Material Data Sheet 1. Further inquiries can be directed to Liqin Zhu, 30819327@nankai.edu.cn.
